# *Drosophila* model systems reveal intestinal stem cells as key players in aging

**DOI:** 10.1111/nyas.15351

**Published:** 2025-04-25

**Authors:** Joung‐Sun Park, Mi Jeong Sung, Hyun‐Jin Na

**Affiliations:** ^1^ Institute of Nanobio Convergence Pusan National University Busan Republic of Korea; ^2^ Department of Molecular Biology Pusan National University Busan Republic of Korea; ^3^ Aging Research Group, Division of Food Functionality Research Korea Food Research Institute Wanju Republic of Korea

**Keywords:** aging, aging marker, *Drosophila*, dysplasia, intestinal stem cell

## Abstract

The intestines play important roles in responding immediately and dynamically to food intake, environmental stress, and metabolic dysfunction, and they are involved in various human diseases and aging. A key part of their function is governed by intestinal stem cells (ISCs); therefore, understanding ISCs is vital. Dysregulation of ISC activity, which is influenced by various cell signaling pathways and environmental signals, can lead to inflammatory responses, tissue damage, and increased cancer susceptibility. Aging exacerbates these dynamics and affects ISC function and tissue elasticity. Additionally, proliferation and differentiation profoundly affect ISC behavior and gut health, highlighting the complex interplay between environmental factors and gut homeostasis. *Drosophila* models help us understand the complex regulatory networks in the gut, providing valuable insights into disease mechanisms and therapeutic strategies targeting human intestinal diseases.

## Graphical abstract

In this review, we discuss the regulation of proliferation and differentiation of intestinal stem cells based on previous research findings, summarize studies on the signaling pathways that regulate these stem cell functions, and describe how aging phenotypes observed in intestinal stem cells due to aging and oxidative stress affect intestinal homeostasis.

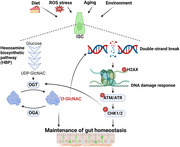

## INTRODUCTION

The intestines are an organ that responds to dietary intake and absorption and responds immediately to external stress.^1^, ^2^ Additionally, aging and various environmental stresses can also upset this balance, triggering an inflammatory response that can lead to impaired gut health.^3^, ^4^ Intestinal stem cells (ISCs) play an important role in maintaining the homeostasis of the intestinal epithelium, which is crucial for overall tissue health and function.^1^, ^5^ The delicate balance between ISC self‐renewal, proliferation, and differentiation tightly controls the rapid regenerative capacity of the intestinal epithelium for tissue regeneration.^6^ Maintaining intestinal homeostasis during rapid renewal requires complex cellular interactions mediated by cytokines, hormones, and commensal microorganisms.^7^ This regeneration capacity is crucial for preserving tissue integrity. However, dysregulation of ISC activity, such as excessive proliferation without proper differentiation, can cause tissue damage, chronic inflammation, and even cancer.^8^, ^9^, ^10^

**FIGURE 1:**
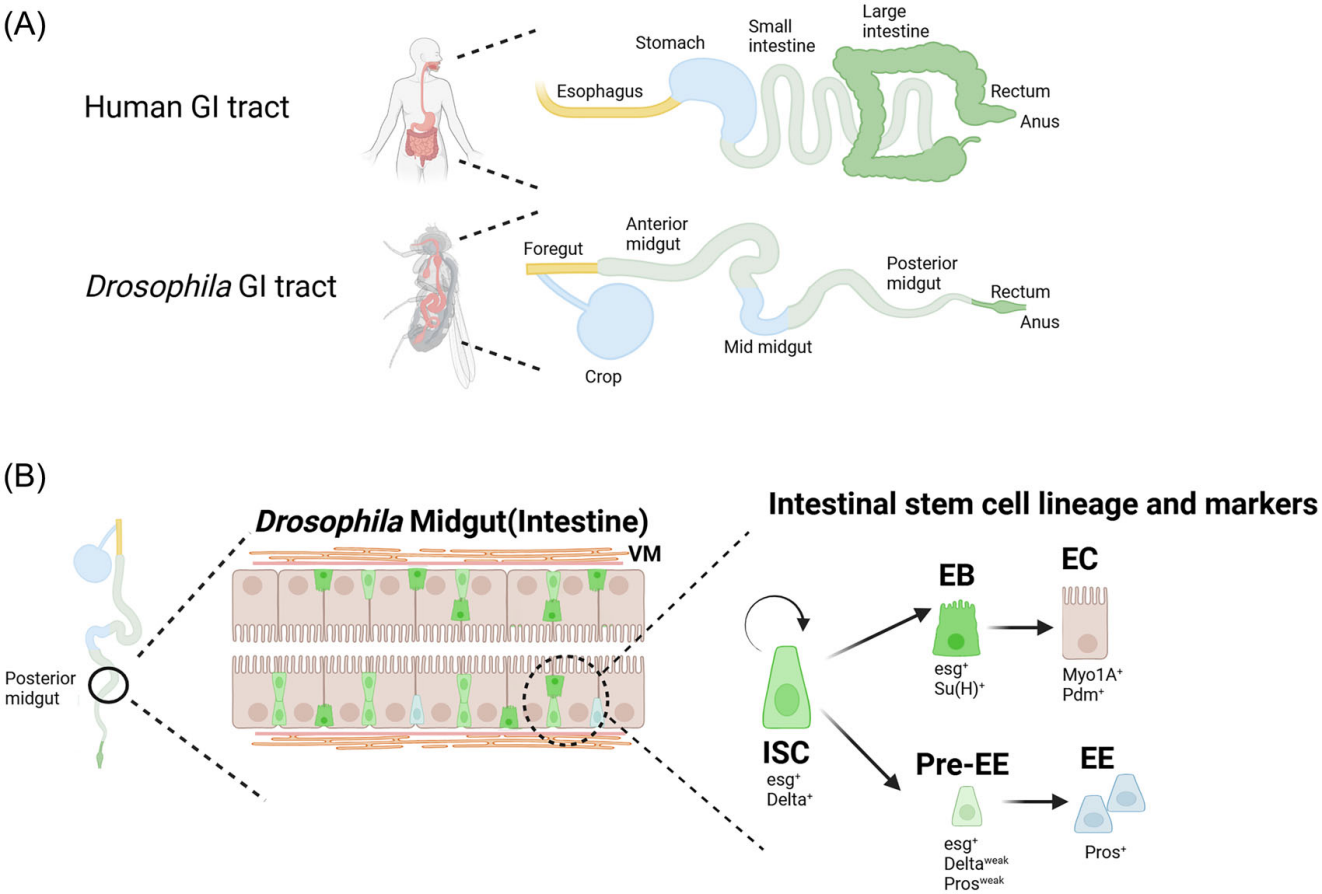
*Drosophila* gut and intestinal stem cell (ISC) lineage. (A) The digestive systems of humans and fruit flies are functionally very similar. The fruit fly foregut corresponds to the human esophagus, the fruit fly crop corresponds to the human stomach, and the posterior midgut of the fruit fly functions similarly to the human small intestine. The midgut of the fruit fly functions like the human stomach. The fruit fly rectum and anus have the same function as the human rectum and anus. The organs that correspond between each other in humans and fruit flies are indicated in the same color. (B) ISCs undergo asymmetric division to generate new ISCs, which maintain the stem cell pool, and precursors called enteroblasts (EBs). The EBs differentiate into absorptive enterocytes (ECs) or secretory enteroendocrine cells (EEs). ISC markers: esg^+^, Delta^+^; EB markers: esg^+^, Su(H) ^+^; EC markers: Myo1A^+^, Pdm^+^; Pre‐EE markers: esg^+^, Delta^weak^, Pros^weak^; EE marker: Pros^+^.

The *Drosophila* intestinal epithelium can be divided into three compartments: foregut, midgut, and hindgut. It is estimated that the intestinal epithelium is completely regenerated only about three to four times during the uniquely short lifespan. Compared to humans, *Drosophila* has a relatively short lifespan, with median and maximum lifespans of approximately 70 and 90 days, respectively, at 25°C.^11^ This rapid regenerative cycle allows study of the response of the intestinal system and ISCs to various environments, pathogens, metabolic stress, and aging, and has served as a model to explore age‐related diseases.^12^ The basic structure of the digestive tract in humans and *Drosophila* includes essential regions of the digestive tract such as the esophagus, stomach, and small intestine, which function to ingest and digest food (Figure 1A). In humans and *Drosophila*, the intestines secrete enzymes to break down food and absorb nutrients.^5^, ^13^ *Drosophila* secretes digestive enzymes in the stomach to facilitate digestion, whereas humans use gastric juices and various digestive enzymes in both the stomach and intestines. Additionally, similar to mammalian intestines, *Drosophila* intestines have conserved cellular pathways that regulate intestinal homeostasis.^13^ As such, the *Drosophila* intestinal model has many advantages, providing similarities and potential implications for understanding human intestinal health and age‐related diseases such as cancer.

In this review, we highlight insights into the regulation of proliferation and differentiation of ISCs based on previous research findings. We then summarize studies on the signaling pathways that regulate these stem cell functions and describe how the phenotypes observed in ISCs due to aging and oxidative stress affect intestinal homeostasis. Finally, we discuss how these discoveries contribute to our current understanding of the pathogenic mechanisms of epithelial dysfunctions that can lead to diseases such as cancer caused by aging or stress.

## NORMAL REGULATION OF ISC PROLIFERATION

### Self‐renewal, proliferation, and differentiation of ISCs

The *Drosophila* intestine is suitable for studying homeostasis of adult stem cells, including their proliferation and differentiation.^14^ Understanding adult stem cells, particularly ISCs, is pivotal to understanding tissue homeostasis.^15^ Adult stem cells regulate tissue function and respond to environmental signals and niches that influence their activity.^16^ The *Drosophila* gut has advanced our knowledge of ISC behavior.^5^, ^14^ In *Drosophila*, ISCs reside within the adult gut epithelium, primarily in the posterior midgut. These ISCs undergo asymmetric division, generating both new ISCs to maintain the stem cell pool and precursors called enteroblasts (EBs). EBs further differentiate into absorptive enterocytes (ECs) or secretory enteroendocrine cells (EEs)^17^, ^18^ (Figure 1B).

The fate of these progenitor cells is tightly regulated by signaling pathways. For instance, Notch signaling promotes EC fate by inhibiting their differentiation into EEs.^19^, ^20^ Conversely, Notch signaling must be turned off or kept low for differentiation into EEs to occur. EE‐specific progenitor cells can undergo mitosis and generate two EEs without undergoing cell division.^21^ EEs play a crucial role in regulating both intestinal and systemic lipid homeostasis. By sensing and responding to dietary lipids, EEs influence lipid absorption, metabolism, and storage, thereby maintaining energy balance. Their function extends beyond lipid regulation, as they also modulate key physiological processes such as food intake, locomotor activity, and overall lifespan.^22^ This underscores the dynamic nature of ISC lineage differentiation in response to physiological demands. Therefore, understanding ISC biology is essential for elucidating the mechanisms underlying diseases influenced by aging and environmental stress.

### Mechanisms regulating ISC proliferation for gut homeostasis

**FIGURE 2:**
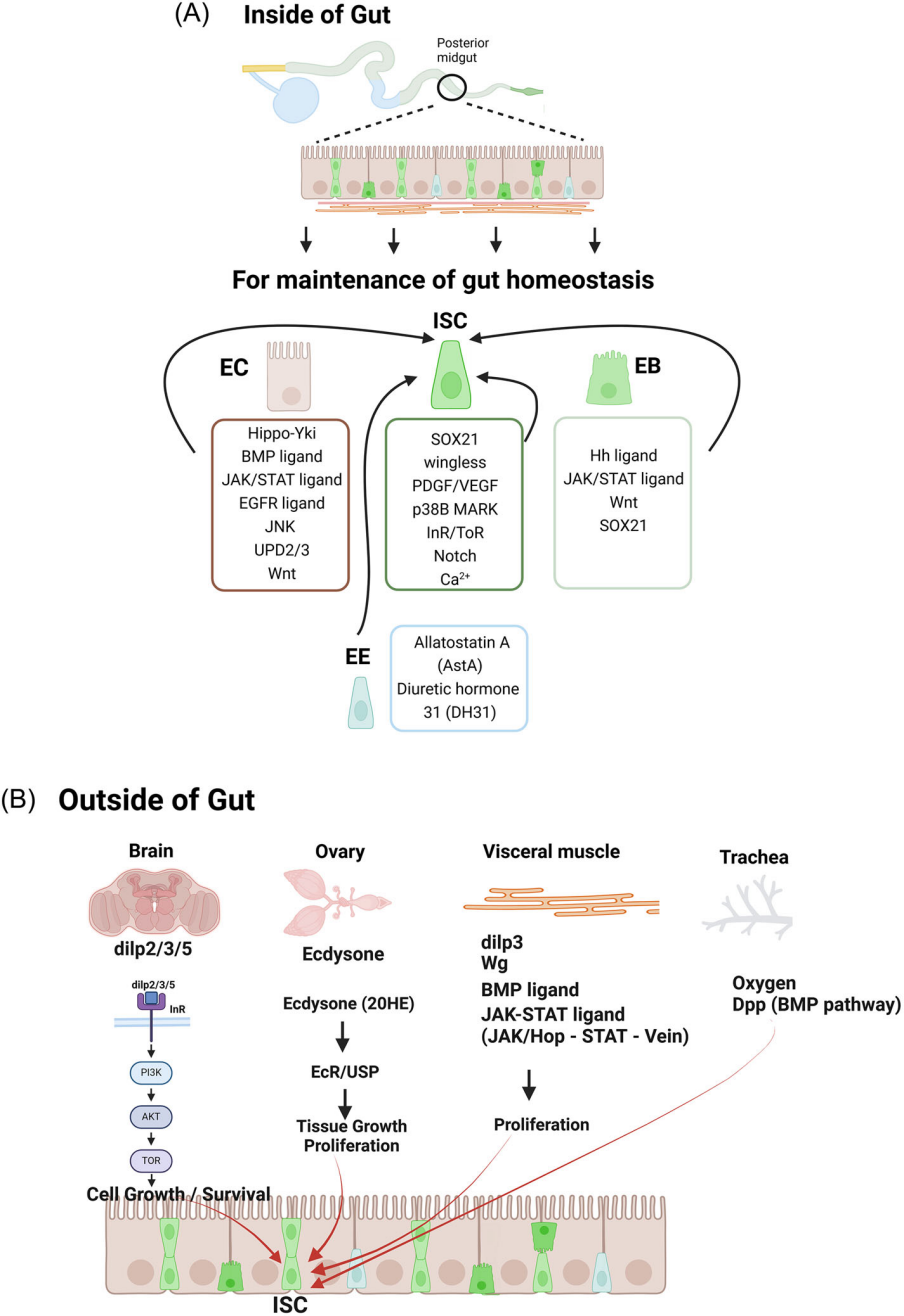
Regulation of intestinal stem cell (ISC) proliferation in normal tissue. (A) Inside the gut, the various cell signaling pathways that influence ISC proliferation and differentiation in *Drosophila* include Hippo‐Yki, bone morphogenetic protein (BMP) ligands, Janus kinase/signal transducer and activator of transcription (JAK/STAT) ligands, epidermal growth factor receptor (EGFR) ligands, c‐Jun N‐terminal kinase (JNK), unpaired (UPD)2/3, and Wnt from EC cells; Hedgehog (Hh) ligands, JAK/STAT ligands, Wnt, and SRY‐box transcription factor 21 (SOX21) from EB cells; and allatostatin A (AstA) and diuretic hormone 31 (DH31) from EE cells. SOX21, wingless (Wg), platelet‐derived growth factor/vascular endothelial growth factor (PDGF/VEGF), p38 mitogen‐activated protein kinase (p38B MARK), insulin receptor/target of rapamycin (InR/ToR), Notch, and the Ca^2^⁺ signaling pathway regulate ISC proliferation. (B) Outside the gut, ISC proliferation can be controlled by ligands or cytokines from tissues other than those of the intestine, such as the brain, visceral muscle, ovary, and trachea. The brain can influence ISC proliferation through various signals, particularly through dilp2/3/5 peptides. Dilp2/3/5 activate InR, AKT, and TOR signaling. Signaling promotes cell growth and regulates survival. The visceral muscle plays a role in controlling the movement of the gut, and it can also release signals that affect ISC behavior. For example, dilp3, wg, BMP ligand, and JAK‐STAT ligand regulates ISC behavior. The *Drosophila* ovary secretes various steroid hormones, particularly ecdysone, which may play a significant role in ISC regulation. In *Drosophila*, the ecdysone receptor (EcR) is expressed in ISCs and differentiated intestinal cells. In the *Drosophila* model, the trachea influences ISC proliferation and differentiation through oxygen supply and specific signaling pathways. EB, enteroblast; EC, enterocyte; EEs, enteroendocrine cells.

Cellular signaling pathways for intestinal development and regeneration are conserved between mammalian and *Drosophila* models.^23^ Some cell signaling pathways that influence ISC proliferation and differentiation in *Drosophila* include the Notch,^24^ Wingless (Wg),^25^ epidermal growth factor receptor (EGFR),^26^, ^27^, ^28^, ^29^, ^30^ Jun‐N‐terminal kinase (JNK),^31^, ^32^ Warts and Yorkie,^33^ target of rapamycin (TOR),^34^, ^35^, ^36^ insulin,^37^, ^38^, ^39^ p38‐MARK,^40^ PDGF/VEGF,^41^ JAK/STAT,^31^, ^42^ Hippo,^43^, ^44^, ^45^ and Decapentaplegic (Dpp; *Drosophila* homolog of bone morphogenetic protein [BMP])^46^, ^47^, ^48^ signaling pathways. Many local, paracrine, and systemic signals and signaling pathways controlling ISC proliferation and differentiation have been identified^29^, ^49^, ^50^ (Figure 2A). ISC proliferation is regulated by various signaling pathways, which can be classified as cell‐autonomous (acting within ISCs) or non‐cell‐autonomous (signals from surrounding cells) pathways. Cell‐autonomous pathways regulate ISC proliferation through intracellular signaling, meaning the signal is processed within the ISC itself. Active Notch signaling in ISCs inhibits proliferation and promotes differentiation.^24^ Conversely, reduced Notch activity leads to ISC overproliferation. Activation of JAK/STAT signaling in ISCs promotes proliferation, and the receptor Domeless (Dome) is expressed in ISCs, where it is activated by Unpaired (Upd) ligands. Wg ligands can be produced within ISCs,^31^, ^42^ leading to self‐activation, and β‐catenin (Armadillo, Arm) activation in ISCs enhances proliferation. EGFR signaling activation in ISCs triggers the Ras/MAPK pathway,^30^ leading to increased proliferation. Non‐cell‐autonomous pathways regulate ISC proliferation through signals originating from neighboring cells such as ECs, EEs, visceral muscle, or damaged tissue. Unpaired (Upd1, Upd2, Upd3) ligands of JAK/STAT signaling are secreted by ECs, EEs, and visceral muscle.^51^ These ligands activate Dome receptors on ISCs, promoting proliferation. Spitz (Vn) and Keren (Krnl) ligands of EGFR signaling are secreted by surrounding ECs and visceral muscle.^51^ These ligands bind to EGFR on ISCs, triggering Ras/MAPK signaling and promoting proliferation. Hedgehog (Hh) ligands, secreted by surrounding ECs, bind to Patched (Ptc) receptors on ISCs, regulating proliferation.^52^ Yorkie (Yki) activation in ECs upregulates Upd3 expression, which then activates JAK/STAT signaling in ISCs, promoting proliferation^33^ (Table 1). ISC proliferation is tightly controlled by both intrinsic (cell autonomous) and extrinsic (non cell–autonomous) signaling pathways, ensuring proper intestinal homeostasis and regeneration. The proto‐oncogene Myc also plays a crucial downstream role by integrating multiple signaling pathways at the transcriptional level to promote optimal ISC proliferation following tissue damage^36^, ^53^ Another transcription factor, SOX21a, acts in both ISCs and EBs to regulate ISC proliferation under normal conditions and promote ISC division in response to stress. This finding highlighted its dual role in maintaining tissue homeostasis.^54^, ^55^ Recently, intracellular Ca^2+^ signaling has emerged as a pivotal regulator of ISC proliferation in *Drosophila* in response to diverse mitogenic signals.^56^, ^57^, ^58^ In ECs, p38 signaling promotes intestinal regeneration by sensing stress and inducing ISC proliferation. ROS generated by the Nox–Ask1–MKK3‐p38 pathway are essential for this response after detergent exposure.^59^ EEs produce hormones such as allatostatin A (AstA) and diuretic hormone 31 (DH31) and regulate local and systemic metabolism, which influences food intake, motor activity, and lifespan.^60^ These findings underscore the intricate interplay between neuronal regulation, hormonal signaling, and nutrient availability in ISC behavior and overall survival, providing insights into therapeutic strategies for human metabolic disorders.^38^ Understanding the roles of these ligands and their ISC‐related interactions provides insights into how stem cell behavior is regulated and how diseases perturb these processes.

**TABLE 1:** Cell‐autonomous and non‐cell autonomous pathways.

Classification	Pathway	Components
Cell‐autonomous	Notch	N receptor (expressed in ISCs)
	JAK/STAT	Dome receptor (activated in ISCs)
	Wnt/Wg	Arm/β‐catenin (activated in ISCs)
	EGFR	Ras/MAPK (activated in ISCs)
	Pvf2	Pvf2 (PDGF/VEGF‐like factor 2) (activated in ISCs)
	InR	InR receptor / ToR (activated in ISCs)
Non‐cell‐autonomous	JAK/STAT	Upd1/2/3 (secreted by ECs, EEs, visceral muscle)
	EGFR	Spitz, Keren (secreted by surrounding cells)
	Hedgehog	Hh ligand (secreted by surrounding cells)
	Hippo	Yki → Upd3 (activated in enterocytes)

### Regulation of ISC proliferation by other organs

ISC proliferation can be controlled by ligands or cytokines from non‐intestinal tissues as detailed below (Figure 2B).

#### Brain

In *Drosophila*, the brain, as part of the central nervous system (CNS), is connected to the ventral nerve cord and is essential for sensory processing, movement control, metabolism regulation, and behavior modulation. The peripheral nervous system (PNS) facilitates sensory and motor communication between the CNS and the body.^61^ The gut is innervated by both the CNS and PNS, with the CNS, including the brain and ventral nerve cord, regulating gut function via descending neural pathways, while the PNS transmits sensory and motor signals between the gut and CNS.^62^ These neural circuits function similarly to those in mammals, where specific neuronal populations and brain–gut hormones regulate homeostatic metabolic changes, thereby influencing feeding behavior and metabolic functions. When these neural and hormonal signaling mechanisms become dysregulated, they can lead to metabolic imbalances in *Drosophila*, akin to conditions such as diabetes and metabolic syndrome in mammals.^62^ These neurons regulate feeding behaviors and metabolic functions, similar to their mammalian counterparts, in which specific neuronal populations and brain–gut hormones control homeostatic metabolic changes.^63^, ^64^ Dysregulation of these mechanisms is implicated in conditions such as diabetes and metabolic syndrome.^65^, ^66^, ^67^ In ISCs, insulin‐like peptides (dilp2/3/5) produced in the brain are crucial regulators,^68^, ^69^, ^70^ demonstrating the direct connection between nutrient sensing and ISC behavior.^68^, ^69^, ^70^ Moreover, manipulating insulin levels can modulate various traits in flies by influencing the insulin/IGF‐like signaling pathway.^71^ JAK/STAT signaling in the gut responds to activated neuronal Hh (Hedgehog) signaling.^72^ Limiting endoplasmic reticulum (ER) stress in ISCs delays stem cell aging, and changes in PGC‐1 activity in high‐turnover tissues such as in the gut may determine mammalian lifespan.^73^

#### Trachea

The *Drosophila* tracheal system is an oxygen delivery network, analogous to the mammalian vascular and respiratory systems.^74^ In *Drosophila*, the trachea influences ISC proliferation and differentiation through oxygen supply and specific signaling pathways. The trachea delivers oxygen to intestinal tissues, and hypoxic conditions (low oxygen levels) significantly impact ISC proliferation. When oxygen levels decrease, hypoxia‐inducible factor‐1α (HIF‐1α) is activated. HIF‐1α regulates transcription factors that promote ISC proliferation and regeneration.^74^ When oxygen levels are sufficient, ISC differentiation increases while proliferation decreases. This suggests that oxygen levels are a key factor in regulating ISC regeneration rates. Bone morphogenetic protein (BMP) signaling plays a crucial role in ISC proliferation and differentiation.^47^ Reduction of BMP signaling in mammalian gut villi leads to over‐proliferation of ISCs, indicating its role in maintaining ISC homeostasis.^75^ Similarly, the role of Dpp in the trachea has been studied in *Drosophila*. Whether the loss of Dpp in the trachea limits ISC proliferation remains unclear.^48^ Some studies suggest that the trachea may regulate BMP signaling within the ISC niche. The trachea may secrete specific BMP‐related factors that influence ISC proliferation. However, further research is needed to determine how BMP signaling in the *Drosophila* trachea correlates with that in mammalian gut villi.

#### Ovary

Sexual dimorphism in *Drosophila* results in physiological and biochemical differences between females and males within various tissues.^76^ In flies, the ovary secretes various steroid hormones, particularly ecdysone, which regulates ISC dynamics through specific receptors and downstream targets.^77^, ^78^, ^79^ Increased ecdysone levels in females can lead to age‐related intestinal dysplasia and tumorigenesis, ultimately shortening lifespan.

In *Drosophila*, the ecdysone receptor (EcR) is expressed in ISCs and differentiated intestinal cells.^77^, ^78^ Ecdysone may regulate transcription factors that control ISC proliferation and differentiation. Changes in ecdysone levels secreted by the ovary could alter ISC proliferation rates. The ovary's function is closely linked to the reproductive cycle, which may also influence ISC regulation. Studies suggest that ISC activity increases during the fertile period. This may be an adaptive response to enhance nutrient absorption to meet the metabolic demands of reproduction.^77^, ^78^, ^79^ Additionally, the sex of the organism profoundly influences gut regulation and proliferation. It acts intrinsically within ISCs and possibly ECs, impacting intestinal size, stem cell proliferation, stress responses, and structural changes during aging,^32^ underscoring the complex interplay between cellular signaling pathways and intrinsic developmental programs governing ISC behavior.^77^, ^80^, ^81^ Therefore, the physiological state of the ovary may act as a key regulator of ISC proliferation and regeneration. Recent studies suggest that both the trachea and ovary can influence ISC proliferation, though the exact mechanisms remain unclear and require further investigation.

#### Muscle

*Drosophila* visceral muscle (VM) impacts digestion, stem cell homeostasis, and intestinal development.^82^ In *Drosophila*, several signaling pathways are mediated by ligands produced by epithelial cells and the surrounding VMs,^82^ maintaining tissue homeostasis and responding to environmental cues. The Wg (Wnt) ligand is produced by the VM and progenitor cells; Wg signaling is essential for ISC proliferation and differentiation.^83^, ^84^, ^85^ The JAK/STAT ligands include Upd1, Upd2, and Upd3.^86^, ^87^ Upd1, expressed by ISCs, EBs, and the VM, regulates ISC proliferation, while Upd2 and Upd3 are expressed by epithelial cells (ECs/ISC/EBs and ECs, respectively) and are critical for modulating ISC behavior and stress responses. EGFR ligands include Vein, Keren, and Spitz. Vein^29^, ^85^ is secreted by the VM and activates the EGFR signaling pathway in the gut. In contrast, Keren and Spitz^85^, ^88^ are induced by ECs and EBs, respectively, and regulate ISC proliferation and differentiation via EGFR pathway activation. Dilp3 is locally produced in the posterior midgut VM, activating ISC division in response to refeeding, thereby directly linking ISC proliferation to food availability.^89^ Expression of these factors can increase due to aging or oxidative stress, leading to the overproliferation or abnormal differentiation of stem cells. These findings provide potential implications for human gut health and therapeutic strategies.

## STEM CELL PROLIFERATION IN AGING

### Dysplasia and aging

**FIGURE 3:**
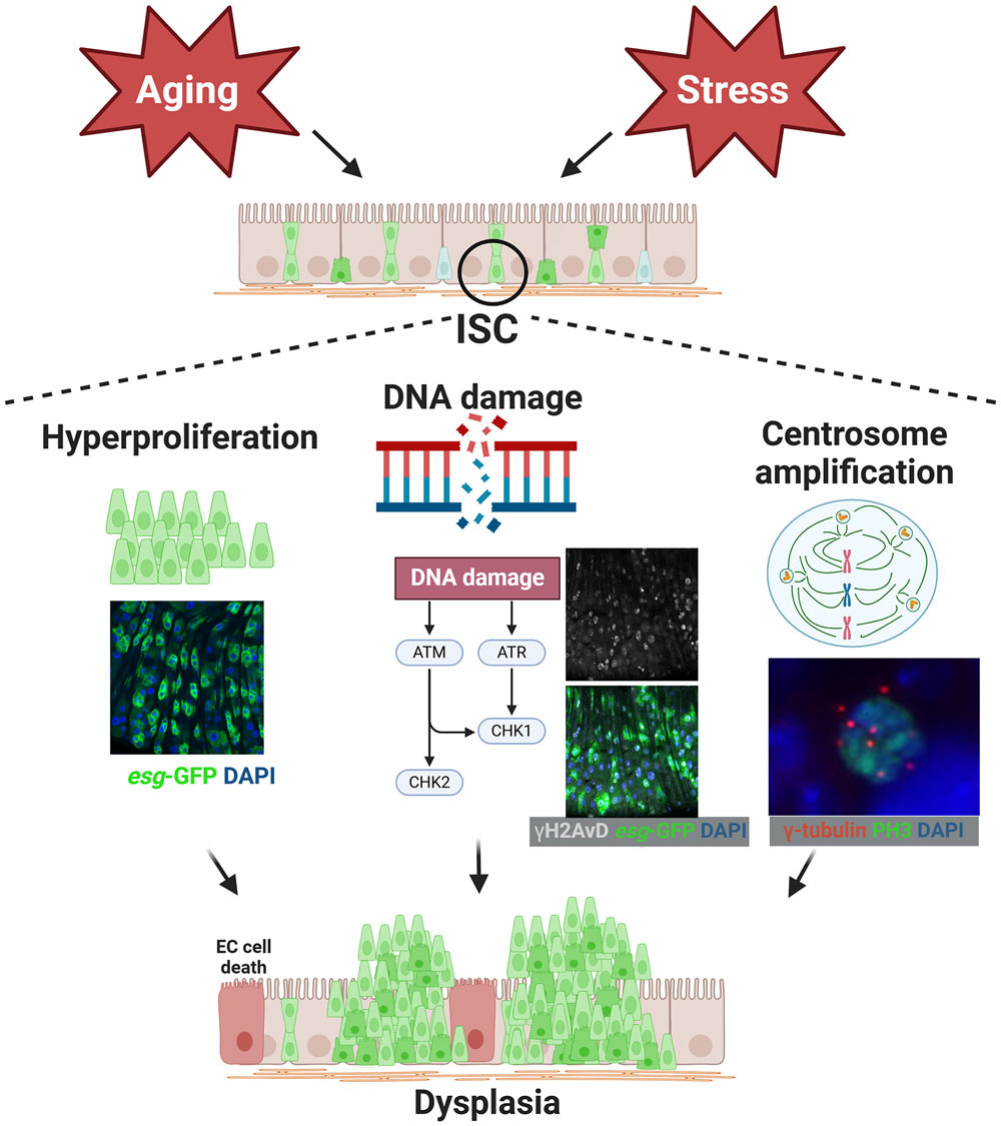
Age‐related markers and the *Drosophila* intestine. As organisms age or experience oxidative stress, various cellular processes can become impaired, including those involved in maintaining proper stem cell function. In the case of intestinal stem cells (ISCs), aging or oxidative stress can lead to significant cellular changes, such as centrosome amplification and DNA damage accumulation, both of which disrupt normal stem cell behavior and ultimately affect tissue function. The combination of aging or oxidative stress, centrosome amplification, and DNA damage in ISCs leads to excessive and unchecked cell proliferation, paired with abnormal differentiation. This disrupts the normal tissue structure and function of the intestine, contributing to the development of a dysplastic phenotype, which could eventually progress to more severe pathologies such as cancer.

The ability of stem cells to proliferate and differentiate is affected by aging. The intestinal aging phenotype is characterized by gut dysplasia resulting from ISC hyperproliferation and abnormal differentiation.^90^ Dysplasia is a precursor to cancer, indicating a breakdown in normal tissue architecture and function.^91^ In aging flies, ISCs hyperproliferate, leading to the accumulation of abnormal cells expressing both stem and progenitor cell markers^30^, ^32^, ^41^ (Figure 3). Age‐related intestinal dysplasia is driven by increased JNK and/or PDGF/VEGF signaling.^30^, ^32^, ^41^ Additionally, chronic activation of JNK and JNK‐mediated cytokine/JAK/STAT signaling can induce similar dysplastic phenotypes in response to oxidative stress or infections.^31^ These dysplastic changes can compromise epithelial barrier function and are strongly correlated with the lifespan of *Drosophila*.^92^ Ultimately, this dysregulation may cause dysplasia and cancer of the intestinal tract.^91^, ^93^

Centrosomes are the primary microtubule‐organizing centers of mammalian cells, playing crucial roles in organizing the mitotic spindle to promote cell division, guiding cell movement through microtubule dynamics, and positioning intracellular organelles to maintain cell polarity.^94^ Since centrosomes are vital for cell proliferation, differentiation, and migration, excess centrosomes often appear early during tumorigenesis and aging.^95^ Centrosome amplification promotes tumor metastasis, making it a potential marker of tumorigenesis.^96^ In *Drosophila*, centrosome amplification can lead to the asymmetric division of stem cells, resulting in an expanded and proliferative stem cell population, ultimately contributing to dysplasia.^97^

Cell cycle arrest—particularly in the G2 phase—can occur due to aging‐ and stress‐related DNA damage and is involved in centrosome amplification.^98^ In high‐turnover tissues such as the intestines, DNA damage accumulates with age in resident stem cells that undergo repeated regeneration.^99^ This supports the hypothesis that centrosome amplification increases with age, potentially leading to tissue aging, decreased lifespan, and age‐related diseases such as cancer. Overexpression of PVR, EGFR, and AKT in ISCs/EBs can induce mitotic ISCs with supernumerary centrosomes, contributing to age‐related changes in the gut, such as hyperproliferation and dysplasia.^100^ Thus, maintaining centrosome homeostasis in tissue‐resident stem cells—especially in high‐turnover tissues like the intestines—is essential for preserving tissue homeostasis, regeneration, and longevity. Studying centrosome amplification in tissue‐resident stem cells is crucial for the prevention and treatment of cancer and other age‐related diseases. Understanding these processes can elucidate the pathophysiology of intestinal diseases and lead to targeted therapeutic strategies.

### Genomic instability and aging

ISC regulation is critical for maintaining epithelial homeostasis and ensuring proper responses to tissue damage and stress. Studies on age‐related dysfunctions indicate that epithelial disorders often result from dysregulated ISC activity and function.^12^ Age‐dependent accumulation of DNA damage is a hallmark of aging in mammalian tissues.^101^ This accumulation impacts the development of age‐related diseases in humans, including neurodegenerative diseases,^102^ bone marrow failure,^103^ heart disease,^104^ hypertension,^105^ and type 2 diabetes.^106^

**FIGURE 4:**
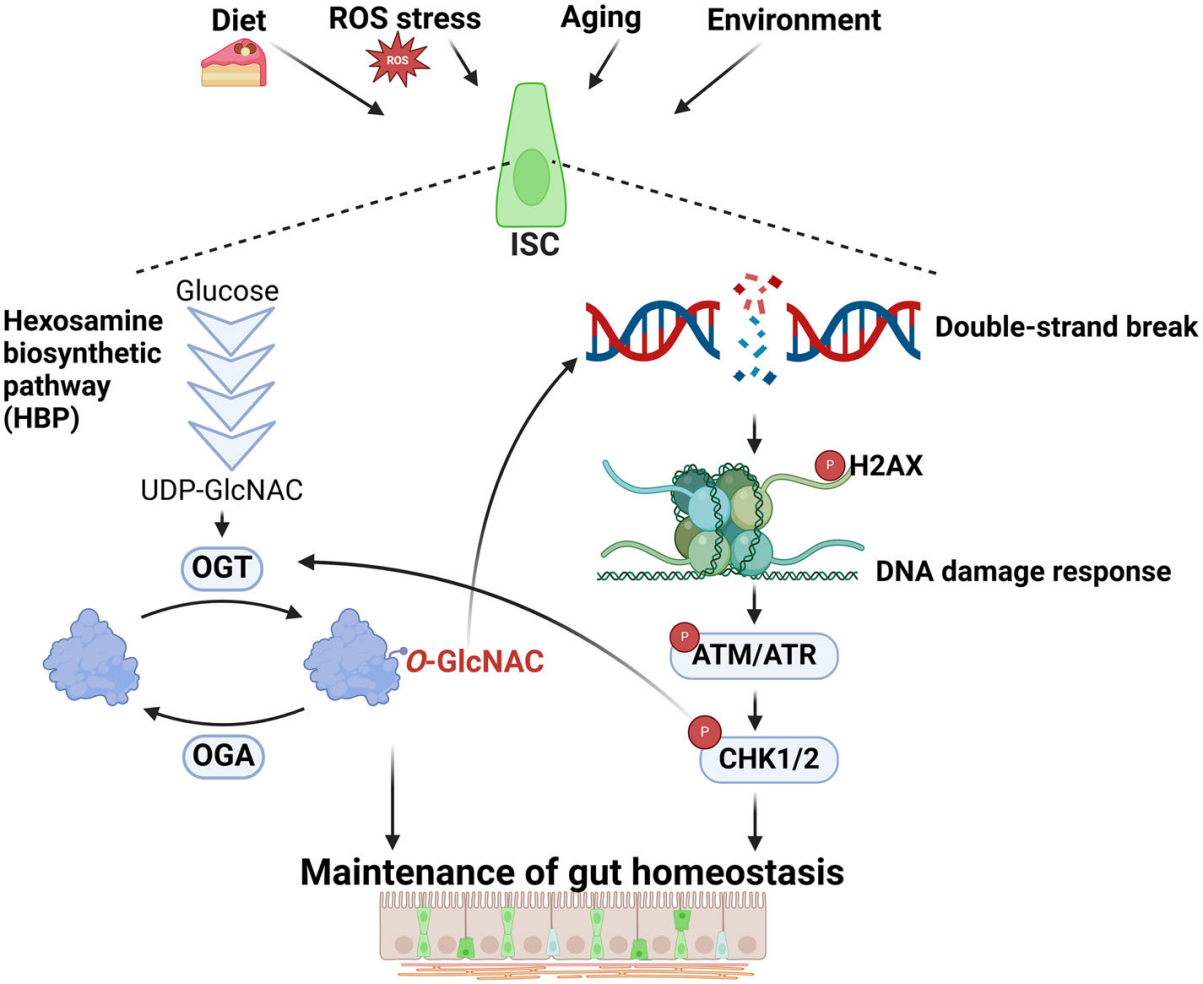
Nutrition and DNA damage response in *Drosophila*. *O*‐GlcNAcylation is regulated by diet, reactive oxygen species stress, aging, the environment, and development. The enzyme that adds *O*‐GlcNAc to the serine and threonine residues of intracellular proteins, *O*‐GlcNAc transferase (OGT), utilizes the product of the hexosamine biosynthetic pathway, UDP‐GlcNAc, as a sugar donor. The *O*‐GlcNAc modification is hydrolyzed by *O*‐GlcNAcase (OGA), creating a dynamic cycle of addition and removal. Nutrient‐dependent *O*‐GlcNAcylation enhances the DNA damage response by regulated activities of OGT and CHK1/2 in ISCs. Age and oxidative stress, particularly ATR and ATM levels, are DNA damage response factors.

Although DNA damage may not directly cause these diseases, its gradual accumulation is correlated with increased cell turnover, which can exacerbate disease progression. In ISCs, various stressors such as ROS (reactive oxygen species), ionizing radiation, and repeated cell cycle activity contribute to DNA damage.^107^ Aging‐related genomic instability in ISCs is marked by increased levels of indicators such as gamma‐H2AX and 8‐oxoguanine, both of which reflect DNA damage accumulation.^100^ Interestingly, DNA damage response factors, particularly ATM (ataxia telangiectasia mutated) and ATR (ATM and RAD3‐related) are crucial to maintaining ISC function.^108^ ROS accumulation during aging induces cell death in differentiated ECs, while H2AX accumulates in ISCs. Under these conditions, ATR and ATM influence the balance between cell death and proliferation in differentiated and stem cells, respectively.^108^ Hyperplasia and increased centrosome amplification have been observed in aged or ROS‐treated intestines and can be used as aging markers for ISCs^100^, ^108^, ^109^ (Figures 3 and 4).

Understanding the molecular pathways underlying the DNA damage response and repair mechanisms in the context of aging and stress is essential for developing targeted interventions to prevent or treat gastrointestinal (GI) disorders associated with epithelial dysfunction and cancer progression. These findings highlight the dual role of the ROS‐induced DNA damage response in stem cells versus differentiated cells, emphasizing the complex interplay between oxidative stress, aging, and intestinal homeostasis. Aging accelerates DNA damage accumulation, and ATM and ATR deficiency shortens lifespans, indicating that a balanced DNA damage repair mechanism is essential.

Heterochromatin is composed of tightly condensed DNA and histone proteins.^110^ Chromatin changes accelerate aging and age‐related diseases.^111^ Specifically, loss or redistribution of heterochromatin is critical for aging, as it induces genomic instability.^112^ H3K9me3 is a binding site for heterochromatin protein 1 (HP1), which plays a role in heterochromatin formation, stabilization, and propagation.^113^ Reduced H3K9me3 levels are also associated with mammalian cell aging.^114^ Age‐related loss/destabilization of H3K9me3/HP1 has also been reported in *Drosophila*.^115^ The *Drosophila* midgut system revealed age‐related loss of heterochromatin stability in differentiated ECs through condensed H3K9me3/HP1 foci, indicating an impact on the age‐related phenotype of ISCs.^116^ Additionally, this study suggests that oxidative stress and AKT/TOR signaling may cause the age‐related loss of heterochromatin stability in ECs.^116^ Therefore, chromatin stability is important for maintaining intestinal homeostasis. Ultimately, understanding the mechanisms driving age‐dependent genomic instability may elucidate the pathogenesis of various age‐related diseases and lead to strategies to mitigate their effects on human health (Figure 3).

### Diet and aging

Calorie restriction (CR), which reduces dietary intake to below energy requirements while maintaining optimal nutrition, is a nutritional intervention with anti‐aging potential.^117^ Given the structural similarities between *Drosophila* and mammalian intestines, currently available powerful genetic tools make *Drosophila* models excellent for studying the impact of this diet on aging. Food abundance partially alters the ISC niche via insulin production.^118^ The intestinal epithelium directly interacts with nutrients and metabolites, which influences lifespan and physiological processes in response to CR and dietary variations.^119^, ^120^ The hexosamine biosynthesis pathway is activated in response to food abundance, leading to a metabolic switch akin to the Warburg effect. This metabolic shift enhances insulin receptor signaling and increases ISC division and overall gut growth.^121^ High glucose levels promote DNA damage accumulation, increase *O*‐GlcNAc levels, and cause hyperproliferation.^122^ *O*‐GlcNAcylation is a nutrient‐driven post‐translational modification associated with cellular stress response.^123^ The enzyme that adds this monosaccharide to the serine and threonine residues of intracellular proteins, *O*‐GlcNAc transferase (OGT), utilizes the product of the hexosamine biosynthetic pathway, UDP‐GlcNAc, as a sugar donor.^123^ *O*‐GlcNAc is hydrolyzed by *O*‐GlcNAcase (OGA), creating a dynamic cycle of addition and removal.^124^ Various cancer types, notably breast, prostate, colon, lung, pancreatic, and colorectal cancer, have upregulated *O*‐GlcNAc levels.^125^, ^126^, ^127^, ^128^ Interestingly, nutrient‐dependent *O*‐GlcNAcylation further exacerbates ISC proliferation, underscoring the intricate link between metabolism, nutrients, and ISC activity.^122^, ^124^, ^129^ In addition, *O*‐GlcNAcylation enhances DNA damage response through the regulation of OGT and CHK1/2 in ISCs.^122^, ^124^ Induction of *O*‐GlcNAcylation in ISCs, influenced by aging and ROS, also promotes ISC hyperproliferation and contributes to intestinal dysfunction^122^ (Figure 4). In response to varying metabolic states and nutrient availabilities, *Drosophila* modulates intestinal growth to adapt to its environment, illustrating stem cell homeostasis in adapting to nutritional cues. Such regulation relies heavily on glycolysis to provide essential metabolites for growth while minimizing the generation of ROS and replicative stress. These insights highlight how the intestine can adjust ISC activity to accommodate organ growth in response to food availability and metabolic cues.

### Anti‐aging drugs

Besides its similarities to human tissues, the *Drosophila* model system is highly suitable for drug screening because of its short lifespan, rapid developmental cycle, and the availability of powerful genetic tools and high throughput methods. Based on these advantages, numerous studies have demonstrated the anti‐aging effects of rapamycin,^130^, ^131^ beta‐hydroxybutyrate,^132^ vitamin D,^133^ and resveratrol.^134^ Additionally, some drugs have anti‐cancer or anti‐aging roles by regulating ISC homeostasis. For instance, metformin reportedly functions as an anti‐aging drug by regulating aging markers, such as excessive proliferation, centrosome amplification, and DNA damage, in the aged intestine.^135^, ^136^, ^137^ Recent evidence suggests that the *Drosophila* model system may be more effective than other model systems for discovering kidney stone–inducing agents and laxatives.^138^ Thus, *Drosophila* serves as an excellent model for drug discovery aimed at treating human diseases.

## CONCLUSION

ISC regulation is essential for intestinal homeostasis. Through complex networks, ISCs adapt to physiological needs, such as nutrient availability and stress responses, ensuring efficient tissue repair and regeneration. Moreover, age‐related studies have highlighted the impact of DNA damage and oxidative stress on ISC function, demonstrating that these factors contribute to age‐related diseases and dysplasia. Age influences ISC regulation and affects both gut health and systemic physiology. *Drosophila* models have deepened our understanding of ISC biology and revealed potential implications for human gastrointestinal health and disease. Uncovering these molecular mechanisms and interactions can help develop targeted therapeutic strategies to manage intestinal diseases and inhibit aging.

## AUTHOR CONTRIBUTIONS

**Joungsun Park**: Writing. **Mi‐Jeong Sung**: Funding. **Hyunjin Na**: Editing, writing, created, and conceptualization.

## CONFLICT OF INTEREST STATEMENT

The authors declare no conflicts of interest.
